# Supplementary material for: Are carbon emissions trading and green financial instruments synergistic? -Comprehensive quantitative research based on content analysis
Source: PLoS One. 2024 Mar 7;19(3):e0298601. doi: 10.1371/journal.pone.0298601 (PMC10919601; doi:10.1371/journal.pone.0298601)
Supplement: S1 Data — (ZIP) [file pone.0298601.s001.zip › Data/地级市Z_F工作报告.zip!/║ú─╧/╚2╤╟╩╨/└┤╫╘╣1⁄2╓┌║┼í╛┬φ┐╦╩2╛▌═°í┐_2007.docx]

各位代表：

现在，我代表市人民政府向大会作政府工作报告，请予审议，并请市政协委员和列席会议的各位同志提出意见。

过去四年工作回顾

2003年以来，在省委、省政府和市委的正确领导下，在市人大、市政协的监督和支持下，本届政府紧紧团结依靠全市人民，以科学发展观为指导，创造性地做好各方面的工作，有效推进了国际性热带滨海旅游城市的建设，圆满完成了市四届人大一次会议确定的各项任务。

一、综合经济实力显著增强。预计2006年全市生产总值完成108.9亿元（含农垦，未经省统计局评估，下同），首次突破100亿元，同比增长14.5%，四年年均增长13.4%，人均生产总值突破2万元大关；其中，一、二、三次产业年均分别增长10.5%、19.8%、12.6%。地方财力大大增强，2006年全市地方财政收入完成10.09亿元，首次突破10亿元大关，同比增长38.1%，四年年均增长31.8%。四年来累计完成固定资产投资186.9亿元，其中2006年完成投资70.8亿元，同比增长40.4%。金融形势稳定，截止2006年底，全市金融机构本外币存款余额160.6亿元，比2002年末增长133.3%；各项贷款余额75.6亿元，比2002年末增长141.7%。

二、产业结构进一步优化。旅游业等传统优势产业发展迅速，商贸服务业逐步壮大，文化产业初现端倪，全市三次产业结构从2002年的32.8：18.9：48.3调整为2006年的28.5：23.5：48。旅游产业方面，《三亚市旅游发展总体规划》编制即将完成，喜来登度假酒店、希尔顿度假酒店等一批高档酒店以及亚龙湾红峡谷高尔夫球场、南山海上观音、大东海酒吧长廊等一批旅游项目相继建成，南山文化旅游区、大小洞天和天涯海角创国家5A级景区工作进展顺利，以打击“四黑”为重点的旅游市场系列化专项整治取得良好成效，旅游宣传促销力度不断加大，国际旅游业发展迅速，转型增效战略取得明显成效。2006年全市旅游饭店共接待过夜游客454.9万人次，同比增长11.9%；旅游总收入65.4亿元，同比增长27.5%；四年年均分别增长13.1%、23.2%。2006年接待境外游客38.8万人次，同比增长83.4%；旅游外汇收入1.7亿美元，同比增长129.1%；四年年均分别增长22.2%、39.5%。农业方面，大隆水库枢纽工程建设接近尾声，半岭、汤他等58宗水库除险加固工程和宁远河南干渠等项目已完成，完成了12个五千亩以上田洋的整治工作，农业生产条件进一步改善；重点实施了无公害农产品基地建设、“十头万户”养猪、兰花示范种植等亮点工程，南繁育种、热带高效农业、畜牧业、海洋渔业等发展迅速；不断加大科技兴农力度，全市发展瓜菜花卉设施农业5万亩；2006年农林牧渔业总产值38.7亿元，同比增长13.4%，四年年均增长9.6%。工业方面，出台了加快新型工业发展的有关政策，加大了工业园区规划建设力度，扶持了绿色农副产品加工、水产品加工、环保建材等一批新型工业。2006年全市工业总产值44.7亿元，同比增长14.3%，四年年均增长9.3%；其中，规模以上工业总产值42.3亿元，年均增长11.4%。企业效益不断提高，2006年全市规模以上工业企业利润总额4900万元，四年年均增长21.6%。房地产业方面，坚持适度、健康发展的方针，加大了土地资源储备、运营力度，目前土地储备总量达到1.8万亩，全面推行国有土地使用权“招拍挂”出让制度；房地产累计完成投资71.6亿元，年均增长66.2%；商品房销售面积213.1万平方米，年均增长30%；销售额达到98.6亿元，年均增长47%。商贸服务业方面，夏日百货、一方百货、明珠购物广场、万福隆超市等大中型商场和超市先后建成投入运营，“万村千乡市场工程”试点工作推进顺利，城乡流通网络不断完善，具有旅游特色的商业网点体系初步形成；商贸经济蓬勃发展，2006年全社会消费品零售总额25亿元，同比增长16.9%，年均增长14.5%。文化产业方面，出台了《关于加快三亚文化产业发展的决定》，明珠影城、时代影城等已建成，体育会展中心正在建设之中，成功举办了第14届金鸡百花电影节暨中国电影百年庆典、2006世界第一大力士冠军赛、TCL高尔夫球精英赛、中国（三亚）国际广告艺术节等大型文化活动，三亚文化产业已具备了加快发展的良好条件。

三、城乡面貌焕然一新。率先在全省完成了近期建设规划和小城镇总体规划；完成了海棠湾概念性总体规划综合方案、海棠湾分区规划以及鹿回头半岛、三亚湾新城、时代海岸、阳光海岸等重点片区的规划设计工作；完成了城市总体规划重大调整，基本完成了旧城区控制性详细规划。不断加大城市基础设施建设力度，完成了两河三岸景观改造、三亚湾路、凤凰路、解放路、新风路和迎宾路等市区道路改扩建工程以及丰兴隆大桥等工程的建设；西环铁路已全线贯通，国际客运港已投入试运行，凤凰机场新航站楼已投入使用；崖城水厂等供水项目、亚龙湾西污水处理站已建成投入使用，城市供水工程、污水管网扩建工程进展顺利；市区主街道电缆下地已基本实现，民用天然气管网已部分供气。积极探索城市拆迁安置方式，在切实维护群众合法利益的同时坚决打击违法建设行为，“半拉子”工程已全部处置完毕。以文明生态村为综合创建载体，社会主义新农村建设稳步推进，到目前已建成了169个文明生态村，特别是去年33个文明生态村建设成效显著；农村水、电、路等基础设施进一步完善，全市共修建乡村公路207公里，完成民房改造4207户、“一池三改”沼气池建设10839户、改厕3.6万多户，兴建各类饮水工程312处，改水受益人口达19万多人，村容村貌发生了巨大变化。开展了城市管理年等活动，创建国家卫生城市工作取得阶段性成果，城市管理水平不断提高。不断推进生态市建设工作，全市森林覆盖率达65%，城市建成区绿地率达41.1%，绿化覆盖率达44.8%，人均公共绿地面积达18.9平方米，生态环境质量继续保持全国一流；城市面貌发生了巨大的变化，获得了“中国人居环境奖”、“最佳中国魅力城市”、“全球绿色生态城市”等荣誉。

四、改革开放取得历史性突破。加快了国有经济战略性调整步伐，完成了北亚航空的国有资产重组，组建并控股三亚市国际旅游会展有限公司和三亚市城市建设投资有限公司，成立了三亚中法水务有限公司；市食品厂等八家企业改制退出工作稳步推进，用1000万元打包回购115家企业6.3亿元的金融债务，融资打包回购企业债务试点工作成效显著；积极鼓励和促进非公有制经济发展，使其成为我市经济发展的主导力量。积极探索推行“土地换社保”等征地补偿配套制度，全面完成农村土地确权工作。我市被批准为全省第一个实行综合行政执法试点工作的城市，成立了综合行政执法局，实施“17+5”综合执法模式。农垦经济体制改革、财税体制改革、金融管理体制改革、教育体制改革以及政府机构和事业单位改革也不断取得新进展。大力实施“文化铺垫、活动支撑、品牌牵引、传媒放大、项目推进、市场运作”的城市品牌发展战略，成功举办了南山海上观音圣像开光大典、第53、54、55届世界小姐总决赛、2006三亚国际美食节、首届中国（三亚）国际热带兰花博览会以及黎苗族“三月三”节等重大节庆赛事活动。去年还开展了大规模的“三亚城市名片”征集评选活动，共征集名片150多万条，引起海内外的强烈关注。对外交流十分活跃，先后与兰州市、乌克兰雅尔塔市等国内外城市结为友好城市。新开了三亚――昆明、三亚――圣彼得堡等多条国内外航线，2006年凤凰机场旅客吞吐量达到390.7万人次，同比增长27.3%；意大利“阿兰格拉”号邮轮每5天一个班次抵达三亚，为我市带来大量国际游客。制定了落实重点推进项目领导责任制、重点项目预警机制和项目退出机制，出台了《三亚市关于改善投资环境的决定》，投资环境进一步优化，一大批国内外知名大企业、大集团已经或即将进入三亚创业。2003年以来，全市实际利用外资5.24亿美元。加大了对出口企业技术改造的扶持力度，对外贸易稳步增长，2003年以来实现外贸进出口总值2.03亿美元，其中出口总值1.4亿美元。

五、人民生活明显改善。2003年以来全市新增城镇就业岗位5万多个，共转移农村剩余劳动力近3万人，下岗失业人员再就业人数达12201人，城镇登记失业率始终控制在3%以内。努力完善社会保障制度，五项社会保险的覆盖面不断扩大，切实落实最低生活保障制度，确保了离退休人员养老金按时足额发放；全市已有25万农民参加2007年新型农村合作医疗，参合率达到95%以上；切实维护农民工利益，四年累计解决拖欠农民工工资3329万元。城乡居民收入保持较快增长，2006年全市单位在岗职工平均工资16661元，同比增长18.5%，四年年均增长10.8%；城镇居民人均可支配收入9416元，同比增长13.1%，四年年均增长13.1%；农民人均纯收入3850元，同比增长14%，四年年均增长8.5%；城乡居民储蓄存款余额80.2亿元，比2002年末增长100.5%。

六、各项社会事业蓬勃发展。四年来，社会事业投入累计突破10亿元。国家农业863海南研发基地等项目基本建成，组建了三亚市南繁科学技术研究院，中国三亚农业科学城等项目稳步推进；不断加强科技创新服务体系建设，建立了全市农业科技服务“110”体系；科技进步对经济增长的贡献率由39.2%提高到43.6%。市九小、三亚市一中新区、三亚鲁迅中学、新育才中学、新教师进修学校、三亚学院、三亚航空旅游职业学院等已建成招生；在全省率先完成中小学D级危房改造，率先免除公办学校义务教育阶段学杂费、贫困地区和部分贫困家庭学生的课本费，“两基”成果进一步巩固，职业教育和高等教育发展态势良好，办学质量不断提高。市人民医院门诊综合大楼、市疾病预防控制中心综合楼、中医院新区住院楼以及崖城等镇卫生院病房等项目已建成投入使用，市人民医院医技大楼、外科大楼、农垦三亚医院传染病区等项目建设进展顺利；重大疾病防治工作成效显著，确保了“非典”期间三亚临床和疑似病例零纪录；2006年人口自然增长率控制在9.3‰以内。群众广场文化活动、文化下乡活动和全民健身运动蓬勃开展，崖城孔庙已修复竣工，为创建崖城历史文化名镇打下了坚实基础。社会主义精神文明建设取得新进步，社会治安综合治理成效显著，市场经济秩序继续好转，安全生产工作进一步加强，社会政治保持稳定。国防动员、民兵预备役工作不断进步，拥军优属、拥政爱民活动深入开展，军政军民团结进一步巩固。统计、民族、宗教、人防、防震减灾、工会、侨联、共青团、妇女儿童、科协、老龄、残疾人事业、新闻、档案及修志等方面工作都取得了较大进步。

七、政府自身建设得到加强。积极组织开展“行政优质服务月”、“三优四满意”等活动，大力贯彻实施《行政许可法》，出台了《三亚市人民政府工作规则》等工作制度和工作规范，制定了《三亚市工程建设项目招标投标办法》等规范性文件，取消行政审批项目329项。倡导并践行政府执行文化，成立了市机关效能投诉中心，大力推行首问责任制、岗位责任制和市政府决定事项、市政府领导批示事项跟踪督办制，建立起行政过错追究制度和投诉处理机制，促进了行政服务效能的提高。加大了行政监察和审计监督力度，2003年以来共完成261个审计项目，审计处理和责成纠正各类违规资金5.55亿元，为财政增收节支2.7亿元。依法接受人大监督和政协民主监督，认真办复市人大代表的议案、建议282件和市政协委员提案420件，人大代表和政协委员的满意率达到97%以上；加强同各人民团体的联系，虚心听取各界人士的建议、意见；充分发挥社会舆论监督作用，让社会各界对政府工作进行监督。深入开展反腐败斗争，四年共查办各类违法违纪案件121件，追缴违规违纪款1683.6万元，为国家和集体挽回经济损失3050.6万元。

各位代表，过去的四年，我市经济社会发展取得了令人振奋的巨大成就，这是省委、省政府和市委正确领导的结果，是市人大、市政协、各民主党派、工商联、人民团体监督支持的结果，是驻三亚部队、武警官兵和人民警察积极参与和配合的结果，是广大海内外朋友关心、支持和帮助的结果，是三亚人民开拓创新、团结奋斗、顽强拚搏、辛勤劳动的结果。在此，我谨代表市政府，向所有参与、支持、关心三亚建设和发展的同志们、朋友们表示崇高的敬意和衷心的感谢！

在肯定成绩的同时，我们也清醒地看到，在前进中我们仍面临着许多困难和问题，还有一些让人民群众不满意的地方，主要表现在：一是城市综合实力仍然较弱。目前我市仍处在发展的初中级阶段，工业化水平低、城市化水平低、城乡居民收入水平低、经济总量小的“三低一小”问题仍然较为突出。二是统筹发展任重道远。目前我市城乡差距仍然较大，社会事业和公共设施欠帐较多，尤其农村基础设施和社会事业发展滞后。三是城市建设和管理水平与建设国际性热带滨海旅游城市目标还有较大的差距。城市功能还不够完善，城市管理仍存在盲区，生态环境保护任务十分艰巨。四是就业和再就业形势不容乐观。农村剩余劳动力向城市转移，城镇新增劳动力，下岗失业人员再就业，大中专毕业生人数增多，四股人流的汇合使我市寻求就业的队伍迅速膨胀，劳动力供大于求的矛盾依然突出。五是平安三亚建设任务艰巨。因征地拆迁、土地纠纷、企业改制、交通混乱等引发的社会矛盾和不稳定因素有所增多。六是政府机关作风建设有待进一步加强。行政不作为、行政乱作为和“吃拿卡要”等现象，在个别单位和少数公职人员身上依然存在。对这些问题，我们将予以高度关注，并采取有效措施在今后的工作中努力加以改进。

今后五年工作安排

今后五年是三亚经济社会快速发展、全面提升的阶段，是我市实现跨越式发展的重要战略机遇期。根据省委、省政府的总体要求和市委的部署，今后五年政府工作的基本思路是：以邓小平理论和“三个代表”重要思想总揽全局，全面贯彻落实党的十六大和十六届六中全会精神，坚持科学发展观，按照市第五次党代会的要求和部署，不断加快产业结构优化升级，逐步构建具有三亚特色的产业体系和经济结构，大胆推进体制机制创新，进一步扩大对外开放，加强生态环境保护和城市管理，充分挖掘城市文化内涵，大力提升城市品位，切实维护社会稳定，注重增强发展的协调性，全力打造“实力三亚、活力三亚、美丽三亚、文化三亚、和谐三亚”，推动三亚经济社会又好又快发展。

根据上述要求，综合考虑各方面的因素，建议今后五年我市经济和社会发展的主要预期目标是：

――打造实力三亚。进一步巩固传统优势产业，培育新的经济增长点，逐步构建新的特色产业体系，推动城乡全面发展，促进经济总量快速增长，城市综合实力显著提升。力争到2011年，全市生产总值达到200亿元，年均增长11%（新口径）；人均地区生产总值34000元，年均增长10.2%；地方财政收入翻一番，超过20亿元，年均增长16%；全社会固定资产投资累计500亿元，年均增长13%。

――打造活力三亚。建设创新型城市，加快改革开放步伐，积极申办和创建三亚发展国际旅游经济综合改革配套试验区，创造更加开放、更具活力的体制机制，建设一个充满活力的现代化热带滨海旅游城市。

――打造美丽三亚。生态环境继续保持全国一流，城市品位进一步提高，彰显“美丽三亚、浪漫天涯”的独特魅力。力争今后五年每万元生产总值能耗年均下降2%左右，人口自然增长率控制在9.3‰以内；到2011年，全市森林覆盖率达到68%，城市生活污水集中处理率超过70%，城市垃圾无害化处理率达到100%。

――打造文化三亚。优化三亚文化发展环境，以文化树形象，以文化铸精神，以文化促发展，使文化成为推动三亚进步的精神支柱和强大动力。在发展文化事业的同时，着力打造“六个一”文化精品工程，积极推进文化产业重点项目建设，努力培育和发展文化产业。

――打造和谐三亚。人民生活更加富足，力争2011年城镇居民可支配收入突破16000元，农民年人均纯收入突破5500元；社会保障体系更加健全，社会就业更加充分，城镇登记失业率控制在3%以内；社会事业全面进步，民主法制更加健全，社会保持安定有序，人民群众安居乐业。

实现新一届政府五年任期目标，首先要扎扎实实做好2007年的工作，努力实现经济社会发展的主要预期目标：：全市生产总值增长11%以上（新口径）；地方财政收入增长16%以上；全社会固定资产投资增长20%左右，达到85亿元；每万元生产总值能耗下降2%左右；城镇居民人均可支配收入增长13%以上，农民年人均纯收入增长9%以上；当年新增就业岗位10500个以上，城镇登记失业率控制在3%以内；人口自然增长率控制在9.3‰以内。

经过十几年的开发建设，三亚已成功地完成了从一个滨海小镇向中国首选的度假旅游目的地的角色转变，具备了加快发展的条件和潜力。刚刚闭幕的市委第五次党代会全面总结了过去四年我市改革发展的基本经验，再次提出了今后五年推进三亚发展的“三大抓手”和“三大支撑点”，进一步明确了六大任务，完全符合科学发展观的要求，为三亚的发展指明了方向。今后五年，无论从国际环境、国内环境、省内环境还是三亚自身的情况来看，都为我市加快发展提供了难得的机遇，我们要珍惜和利用好重要战略机遇，勇于迎接挑战，敢于承担历史赋予我们的重大责任，扎实工作，全面推进国际性热带滨海旅游城市建设。今后五年的政府工作，将重点抓好以下五个方面：

一、坚持发展第一要务，全力打造实力三亚

目前三亚仍处于发展的初中级阶段，城乡二元结构明显，我们要通过不断加快产业结构优化调整，增强三亚产业竞争力，推动三亚城乡全面发展，促进综合实力迅速提升。

――推动农村全面进步。以文明生态村为综合创建载体，积极推进社会主义新农村建设，进一步贯彻工业反哺农业、城市支持农村和“多予、少取、放活”的方针，统筹城乡协调发展，促进农业不断增效、农村加快发展、农民持续增收，力争2011年全市80%以上的村庄建成文明生态村。一是大力完善农村基础设施。要进一步加大财政支农力度，加强农业基础设施建设；做好大隆水库枢纽工程收尾工作并尽快发挥效益，进一步加强病险水库除险加固工作，用两年时间对各水库干渠进行全面防渗硬化；将东部赤田中型灌区、中部三亚河中型灌区和西部大隆水库中型灌区渠系联网，初步完成三亚大灌区建设；充分利用新增水源，采用喷灌、微灌等技术，扩大灌区面积至36.2万亩；继续做好农田综合整治工作；以文明生态村建设为契机，加大对农村民房改造、电网、乡村道路、改水改厕、沼气池、环保设施等工程的投入，不断改善农民的生产生活条件，建成一批集中连片、配套设施完善、村容村貌整洁的村庄。二是加快农业现代化进程。要落实信贷支农政策，大力引进外资，拓宽农业资金来源渠道；进一步调整农业产业结构，重点发展南繁育种、热带水果、热带花卉、冬季瓜菜、热带林木等热带高效农业，继续实施“十头万户”养猪工程，积极推进“无规定动物疫病区”项目和无公害生产基地建设；要加大科技兴农力度，力争农机化作业水平达到种植面积的50%，大力推广农业先进实用技术，不断提高农业科技成果转化率和良种良苗覆盖率；积极发展瓜菜大棚种植等设施农业，力争到2011年全市设施农业超过8万亩；进一步健全农村科技服务体系，重点建设农业科技服务“110”智能网络系统和电子农务系统；提高农业产业化水平，着力培育龙头加工企业，大力推进兰花生产基地、三亚市高新农业示范基地等基地建设；不断完善农业市场体系，加强农业信息网络建设，积极开拓岛外市场，搞活农产品流通。三是发展农村公共事业。要积极推进以镇级机构、农村义务教育、财政管理体制改革为主要内容的农村综合改革，大力发展农村公共事业。切实加大教育扶贫力度，从经费、师资、教学设备等方面对农村地区，尤其是贫困地区和少数民族地区加以扶持；规划好农村学校的撤并工作，加快推进寄宿制学校工程；将沼气池建设纳入农村寄宿制学校基建规划，积极探索生态循环型校园建设；对农村义务教育阶段的中小学生免收学杂费和课本费并对山区住宿生实行生活补贴，对进入我市职业中学的贫困山区学生实行“三免一补”；设立贫困学生助学专项资金，建立和完善贫困学生跟踪扶助制度；实行城乡教师定期交流服务制度和毕业生到农村学校定期服务制度，积极改善农村教师的工作和生活条件，适当提高边远山区教师待遇。继续推进镇卫生院和村卫生室建设，实现卫生院“一无三配套”和村村有卫生室的目标，力争新型农村合作医疗覆盖全市农村居民，提高广大农民健康水平和抵御疾病风险的能力，切实解决农民看病难、看病贵和因病返贫等问题。继续推行“土地换社保”，尽快出台《三亚市被征地农民社会保障制度实施办法》，探索既有利于发展又能维护农民利益的征地制度，不断丰富村民文化生活，完善农村民主管理制度。四是促进农民稳步增收。要不断完善银行扶持农户的各种模式，积极稳妥推进农村信用社改革，推行小额信用、农户联保等贷款方式；继续实施良种补贴、购置农机具补贴等政策，建立农民增收减负长效机制，增加农民政策补贴收入；积极发展乡镇企业，培育农村二、三产业，与六镇建设旅游服务基地相结合，高水准地开发文门、水南等乡村旅游项目，培育扶持一批“旅游示范村”、“旅游示范户”；要加快培养新型农民，着力抓好“农村劳动力转移培训阳光工程”，加强对农民的实用农业技术和职业技能培训，继续发展劳务经济，争取五年内培训农民5万人次，实现农村劳动力转移就业3万人，促进农民多渠道增收。

――进一步加快城市发展。要全力加快城镇建设步伐，提升城市容纳能力和综合竞争力，力争到2011年我市城市化水平达到60%左右。一是拓展城市发展空间。要进一步树立规划先行的理念，做好三亚市总体城市设计，通过合理规划把旧城区改造和新城区建设有机地结合起来，积极完善“一城六镇”的城镇发展框架，推进六镇以及农场场部的规划建设，尤其是要加快育才镇基础设施建设，引导和培育沿海带状城镇群的发展，进一步拓展城市发展空间。二是完善城市功能。积极推进凤凰机场扩建、三亚国际客运港、南山货运港、六道湾渔港、西环铁路提速改造、火车站北迁、三亚绕城高速公路以及长途汽车站等工程建设，全力做好配合工作，为西环铁路早日投入营运和东环铁路建设提供保障，尽快实现公路、铁路、港口、航空的有机联动，努力打造区域性交通枢纽，提升三亚区域性中心城市的地位；加快田独至藤桥城市交通快速干道、六道湾海景大道、鹿回头海景大道、东线高速公路海棠湾出口路、梅山高速公路出口路及红沙大桥等工程建设，形成方便、快捷、高效的城市道路交通网络；进一步完善城市给水排水网，继续推进城市电网改造，提高用水用电保障能力；加快天然气输配管网建设，提高天然气供应能力，力争2011年主城区居民天然气覆盖率达到95％以上；进一步完善以光缆为主，以数字微波和卫星通信为辅，覆盖全市城乡的立体化通信基础网络。

――全面提升旅游产业素质。要大力实施旅游国际化战略，增强三亚旅游业的国际竞争力，力争到2011年，全市旅游饭店接待过夜游客突破800万人次，旅游总收入突破150亿元；其中境外游客突破100万人次，旅游外汇收入突破5亿美元。一是要精心培育名牌景区景点。以创建国家5A级景区为契机，配套完善南山文化旅游区、大小洞天景区功能，改造扩建天涯海角等传统景区景点，进一步加强蜈支洲岛、西岛等景区旅游设施和项目建设，特别是要加快海棠湾“国家海岸”的建设步伐，大力提升景区景点档次。二是要精心培育特色旅游产品。深度开发潜水、海底观光、垂钓和海上游等海洋旅游项目，积极促进西沙旅游开放，大力发展游艇经济和会展经济，打造中国一流、亚洲著名、世界知名的高尔夫天堂。三是要精心培育品牌度假酒店。要积极推进三亚悦榕庄、香格里拉度假酒店、丽思卡顿酒店等一批高档酒店的建设，继续积极引进国际著名酒店管理集团，力争五年内五星级标准酒店达到40家以上，形成具有三亚特色的高端度假酒店品牌。四是精心培育一流的旅游服务环境。要大力开发具有地方特色和文化内涵的旅游商品，构建商品类型丰富、服务功能完善、面向国际消费的商业网络，积极推进机场和市区免税店建设，建设好亚龙湾环湖商业街、三亚湾路酒吧美食一条街、商业街旅游工艺品一条街以及解放路综合商业步行街；重点推进时代海岸、阳光海岸等项目建设，打造独具特色的集休闲、娱乐、餐饮、购物于一体的不夜城。要加强旅游行业管理，开展旅游诚信建设，进一步加大旅游市场整顿力度；建立三亚市游客到访中心，不断完善旅游投诉和旅游执法机制，加强旅游市场管理制度建设；加强从业人员素质培训，加快提高其外语水平；加强与外来企业、外来资本特别是国际大旅游集团的合资合作，提升旅游服务整体水平；逐步建立健全国际化的道路标识系统、旅游标识系统、旅游咨询服务系统、旅游紧急救援系统，努力增强处理涉及国际游客的突发事件的能力。五是精心培育高端客源市场。实施“大企业、大航线、大团队”战略，以2007年海南旅游宣传促销年和三亚国际旅游年为契机，进一步加强旅游促销，充分利用国内外知名旅行商的营销网络，大力开拓海内外客源市场；要综合利用好落地签证、免签证等政策，尽量用足用活航权开放政策，开通更多的国际直达航线航班，吸引境外高端游客来三亚度假。

――积极发展海洋产业。要充分利用区位和资源优势，推动海洋产业发展，力争2011年全市海洋产业总产值达到58.2亿元。一是大力发展海洋渔业。以结构调整为主线，构建三场、三湾、一港的海洋渔业发展新格局，建设中国南海渔业基地；限制近海捕捞，加快发展外海和远洋捕捞，逐步实现外海和远洋捕捞规模化、专业化；大力发展工厂化等先进养殖技术，推动海洋养殖由普通品种向名贵海洋珍品方向发展；限制近岸的高位池养殖，发展深海网箱养殖，推广生态养殖，建设一批名、特、优养殖基地和种苗基地；进一步发展水产品加工业，提高产品附加值。二是大力发展临港工业。加快南山货运港和六道湾渔港建设，南山货运港依托深水良港发展大宗海运货物集散、加工以及各种船舶维修制造等临港工业，六道湾渔港重点发展制冰、冷冻、海产品加工、深海动植物资源深加工等临港工业，积极打造临港工业新区。适应国家南海权益保障和能源安全战略要求，充分利用国家开发南海油气的有利时机，争取将油气开发利用和加工基地放在梅山；大力引进国内外有实力的大型企业，重点发展海洋资源研发、石油储备、油气中转运输、石油化工、天然气化工及下游产品加工，打造主体功能完善、生产生活设施配套的中国南海现代化后勤服务基地和重要的油气加工基地。

――努力发展高科技工业。为壮大我市经济实力，提高工业对经济增长的贡献率，必须加快工业的发展步伐。一是要加快工业园区规划建设。理顺工业园区管理体制，创新工业园区发展模式，设立工业园区土地开发公司，推动工业园区自我滚动发展。大力吸引高新技术项目到市（田独）高新技术产业园、市（荔枝沟）工贸园区落户，以规模项目带动工业园区向生物制药、高精电子产品生产、软件开发等高新技术及食品加工、新型建材、旅游工艺品加工、水产品加工、食品等工业方向发展，提升工业科技含量。积极开展与各高等院校、科研机构的挂钩协作，支持三亚同济科技园的建设与发展，建立产学研一条龙的高新技术开发、科研与应用体系。二是要大力发展创意产业。抓紧启动三亚（南山）创意新城的规划开发建设，抓住与中兴产业集团合作开发创意新城的机遇，创新发展模式，形成独具特色的创意产业群，鼓励和吸引高科技企业和人才，打造三亚“硅谷”。

――稳健发展房地产业。房地产业是三亚的优势产业，也是我市经济增长的重要动力和财政收入的主要来源，要充分发挥三亚的生态环境优势，推进房地产业发展。一是加大对土地市场的调控力度。落实土地储备计划，继续按照“土地供应略少于市场需求”的原则控制土地供应总量，进一步释放土地价值，合理配置土地资源，滨海一线用地原则上不再批建房地产项目，坚持全面推行国有土地使用权“招拍挂”出让制度，确保土地交易的公正、公平、公开。二是提高房地产开发质量。严格实行行业准入制度，鼓励以置业和创业为主的房地产业的发展，加快房地产业规模化发展步伐，抓住海棠湾及三亚湾新城开发的契机，拉动房地产投资大幅增长。三是为房地产交易提供便利服务。探索建立由房产部门统一办理商品房分户土地房屋权证的模式，提高房地产办证效率；对普通住房转让契税滞纳金实行较为宽松、有操作空间和弹性的税收征管办法。进一步培育和规范二手房市场，实行一站式办理制，尽快解决房改房和集资房遗留的办证问题，完善市场体系，形成梯度消费模式。尽快制定并出台相关实施办法，规范产权酒店的开发管理。加强对房地产市场的预测、监测，建立预警体系，保持房地产业健康发展。

二、加快改革开放步伐，全力打造活力三亚

未来五年，三亚改革攻坚的任务繁重，对外开放面临的环境更加复杂，我们要不断加快体制创新，构建经济社会持续快速发展的长效动力机制。

――切实抓好政府自身改革。要大力推进政府职能转变，不断提高行政效能。一是加强政府执行文化建设。强化执行意识，改进执行方式，加大执行力度，大兴求真务实之风，不断提高政府工作效率。设立三亚市行政服务中心，积极推行公开办事、“一站式”服务，进一步清理行政许可项目；继续加快电子政务建设，提高政务信息化水平；完善决策目标、执行责任、考核监督体系，切实落实工作过错责任追究制和行政首长问责制，严肃查处行政不作为、乱作为行为；开办三亚政风行风热线栏目，促进政府与社会、干部与群众的互动。二是加快行政管理体制创新。进一步完善行政管理体制，合理划分市、镇政府权责，下放管理权限，增强发展活力；按照国家和省有关要求，继续精简机构和编制，理顺部门分工，明确职能、权限和责任，着力解决互相推诿、扯皮的问题。三是加快法治政府建设。贯彻实施《行政许可法》，深化行政执法体制改革，严格执行行政执法责任制、评议考核制和过错追究制；建立健全重大决策公示、听证制度和政府法律顾问制度，积极做好行政复议工作，充分发挥层级监督职能。四是加强公务员队伍建设。认真组织实施《中华人民共和国公务员法》，加大公务员培训力度，进一步加强廉政建设，努力建设一支高素质的公务员队伍。

――努力改善市场经济环境。要建立公正公平的市场经济秩序，为各类企业提供平等的发展平台。一是全面深化国资国企改革。要按照“整合发展一批”、“改制退出一批”、“关闭破产一批”的方式，分类推进国企改革、改制和重组；努力培育龙头企业，进一步提高国有经济集中度，增强国有经济的控制力、影响力和带动力；加强对企业国有产权转让的监管，保证国有企业改制规范进行；建立国企改革资金专户，切实解决下岗职工养老、保险等问题，盘活土地房产等优质国有资产；建立科学的企业国有资产统计评价体系，不断完善现代企业制度和国有资产监管体制。二是加快推动非公有制经济发展。最大限度地放宽市场准入的行业和领域，为非公有制经济发展创造宽松、公平、良好的市场环境；认真贯彻实施《三亚市人民政府关于鼓励支持和引导个体私营等非公有制经济发展的若干规定》，切实落实各项扶持、奖励政策，加强对非公有制经济发展的产业引导，提高非公有制经济的比重和水平。三是鼓励中介组织发展。建立与国际通行规则相衔接的行业协会运行机制，发挥行业协会在为企业服务、行业自律、行业协调、对外交往等方面的积极作用；积极发展会计师事务所、律师事务所、专利商标事务所等各种市场中介组织，强化监管机制，并引导其建立健全内部管理制度和信用制度，提高自律性和诚信度。

――积极推动其它各项改革。一是深化财税金融体制改革。要继续调整和优化财政支出结构，促进支出向关系民生的领域、新农村建设和基础设施等环节倾斜；要加强财政绩效预算、绩效评价和绩效审计监督，健全政府投资决策责任制；建立政府债务风险监测和预警机制；逐步建立健全事权与财权相匹配的财政体制，切实提高基层政府的公共服务能力。依法强化税收征管，做到应收尽收。支持金融体制改革，建立高效的金融服务和监管体系，推动资本市场合理利用，保持金融形势稳定。二是加快收入分配制度改革。根据国家和省的统一部署积极稳妥地进行公务员工资改革，在清理规范津贴、补贴的基础上，适当提高公务员工资水平；加快事业单位工作人员工资制度改革，建立符合事业单位特点、体现岗位绩效和分级分类管理的收入分配制度；进一步完善企业工资收入分配宏观调控体系，使企业职工收入随企业效益提高而适当提高，退休人员基本养老金随在职职工收入增加而适当增加；落实最低工资规定，逐步提高最低工资标准，依法加大个人所得税征管力度。三是积极支持农垦经济体制改革。要贯彻落实中央和省农垦改革精神，支持境内国营农场理顺农垦经营管理体制，切实提高农垦经济发展水平。

――进一步提高招商引资水平。三亚目前仍然是投资拉动经济增长为主的城市，必须继续实施“大企业进入，大项目带动”的发展战略，全力抓好重点项目建设。一是要大力改善投资环境。认真贯彻执行《三亚市关于改善投资环境的决定》，积极构建诚信体系，将三亚打造成全国投资环境最好的城市之一。理顺项目进入、报建、建设及竣工管理的审批程序，简化并完善投资审批制度，确保项目快速进入并顺利实施；严格实行项目跟踪服务，完善项目风险预警机制，建立健全项目监督机制和退出机制，有效防范和化解投资项目建设风险，提高项目的履约率，杜绝“半拉子”工程；建立投资环境监督评议评价制度，对投资者反映的问题及时处理；坚持先安置补偿、后拆迁的原则，加大对违章建筑的整治力度并积极解决历史遗留问题。二是要加大招商引资力度。切实加强重大项目策划，努力做到策划一批、储备一批、建设一批、见效一批，实现重大项目不间断、投资增势不减缓；强化产业政策引导，鼓励国内外客商投资基础设施建设、农业综合开发、生态环境建设、旅游资源开发和高新技术产业等领域；要全方位拓宽融资渠道，积极向上争取更多的国债资金和政策性银行贷款资金，努力激活社会投资，同时利用好土地储备资金和地方财政建设资金。抓好与香港盈科地产、新世界和上海家化联合体、保利集团等一批大企业的洽谈跟进，以一流的项目和一流的自然环境资源进一步吸引国内外大财团、大公司前来投资，全力加快海棠湾、红塘湾、鹿回头半岛和凤凰岛等重点区域开发建设进度。

――大力加快对外开放步伐。要充分利用我市的产业特色和环境特色，全方位开展对外交流与合作。一是积极申办和创建三亚发展国际旅游经济综合改革配套试验区。要以此为契机，积极建立与国际通行规则接轨的管理体制，努力实现游客的自由出入，货物的自由进出，项目的自由落户，资金的自由流通，为全省乃至全国旅游体制机制创新探索新路子。二是要全面扩大对外开放。要扩大金融、保险、商业、旅游、会展、科技、教育、文化等领域的对外开放，积极开展与中国――东盟自由贸易区、泛珠三角、长三角、环渤海等区域的交流合作，力争在更大范围、更宽领域和更高层次上参与国内外经济技术合作，创造条件吸引国内外知名企业和公司在三亚设立总部或研发基地，积极发展总部经济。三是要扩大对外贸易。要实施科技兴贸战略，继续加大对出口企业技术改造的扶持力度，支持和培育产品竞争力强、外部市场好的地方龙头企业，增加出口产品的附加值；要重点抓好反季节瓜菜、水果、热带花卉、海水产品等热带优势农副产品及其深加工产品，实施出口品牌战略。力争今后五年实现外贸进出口总值4.67亿美元，其中出口总值3.2亿美元。

三、加强生态环境保护，全力打造美丽三亚

要按照国际性滨海旅游城市的定位，以塑造美丽三亚的城市形象为目标，坚持抓好生态环境保护，促进人与自然和谐发展，进一步提升城市品位，早日把三亚建成“美丽之都”。

――建设环境友好型社会。在发展中始终坚持不污染环境、不破坏资源、不搞低水平重复建设的“三不”原则。进一步加快生态市建设步伐，高标准建设热带森林、红树林、珊瑚礁等自然保护区，重点保护分布于中北部的热带天然林、沿海防护林和红树林，一般地区通过加强生态建设和产业引导减轻生态环境压力；加强生态绿地系统建设，构筑城市防护绿地系统、公路林网和农田林网绿化系统；力争到2011年，城市建成区绿地率达到50.4%以上，绿化覆盖率达到54.1%以上，人均公共绿地面积达到27.6平方米以上；查路补树、查绿补花，认真解决有绿无荫、有绿无花等问题；加强海洋综合管理，建立健全海洋综合管理体系，加大海洋环境监察和整治工作力度，实现海域生态系统、重点入海污染物排放总量和海洋生态环境的有效监控，加快对三亚湾海滩泥化和海岸侵蚀等的治理；积极推进主城区污水管网配套工程以及海坡、鹿回头半岛、海棠湾开发区等片区污水管网和污水处理项目，提高城市污水收集率、处理达标率以及城市生活垃圾收集、运输、处理水平，将垃圾和污水处理作为今后几年生态建设的一号工程切实抓好抓实，力争3―5年内城市垃圾无害化处理率和水质达标率达到100%；合理布局和建设城市公厕，加大对噪声、汽车尾气等污染的监管和治理力度；控制化肥、农药的施用量，全面推广使用有机肥及绿色农药、化肥，积极发展绿色、无公害、有机农产品。

――建设资源节约型社会。建立健全资源合理开发利用体系，推行资源的资产化管理，确保资源可持续产出；加大对土地开发的监管力度，提高土地利用效率；积极推进海域使用权公开招标、拍卖试点工作，努力提高海域资源利用效益；积极发展循环经济，提高能源利用效率，大力推进节能、节水、节地、节材；加快推行节能建筑设计标准，优先发展和完善城市公共交通系统；提高生活垃圾分类收集和综合回收利用的比率；积极推广利用太阳能供电、供热、供冷、照明；结合新农村建设推广农村地区沼气利用，力争五年内新建农村沼气池1.7万个以上，大力发展沼气系列生态农业；严格保护已划定的福万、水源池、赤田、大隆水库的饮用水源保护区，实现水资源的可持续利用，积极推进中水回用设施建设，打造节水型城市。

――建设“美丽之都”。要突出城市个性，提升城市品位，不断提高城市管理水平，优化城市环境，把三亚早日建成人居环境世界一流的城市。一是提升城市品位。要将城市建设与旅游产业发展相结合，进一步突出热带滨海旅游城市的风格，精心美化城市景观，强化风景旅游城市的功能；积极推进三亚河、临春河景观工程等项目建设，通过多姿多彩、和谐有序的建筑设计，突出海、天、山、河四位一体的城市特点，精心打造“城在山中、城在水中、城在绿中、城在花中”的热带滨海城市景观。二是强化城市管理。要积极推进基础设施投融资、维护保养、收费体系等制度改革，加快市政公用设施市场化步伐，提高运营管理效率；进一步加快城市管理标准化进度，不断推进城市管理综合行政执法试点工作和社区建设工作，推动城市管理重心下移，构建科学运行、高效管理、适度超前的新型城市管理模式；加强城市规划监管力度，继续依法严厉打击违法建设，用五年时间基本完成“城中村”综合整治开发；创造条件对农贸市场进行升级改造，五年内基本实现农副产品超市化经营；积极建设智能交通监控系统，继续深化交通秩序综合整治，坚决取缔非法营运的“摩的”、“黑车”和报废车，大力整顿交通营运市场，坚决遏制重特大道路交通安全事故的发生；加强城市环境卫生管理，不断优化城市环境；积极开展城市管理和法律法规的宣传活动，不断提高居民的城市意识和文明程度，全面加强城市管理。

四、挖掘城市文化内涵，全力打造文化三亚

文化是一个城市的灵魂，我们必须充分挖掘城市文化内涵，并在推动传统文化事业发展的同时通过吸引文化产业重点项目、打造文化精品和举办大型活动等手段，推动三亚文化产业发展，把三亚建设成为海南文化产业的重要基地。

――充分挖掘城市文化内涵。要进一步加强三亚文化研究工作，加大文化挖掘的力度和深度，积极开发三亚独特的生态文化、海洋文化、历史文化、少数民族文化以及长寿文化等，建立文化多元化综合体系。特别是要充分利用黎、苗、回等少数民族文化资源，建设一个中国一流的民俗文化村；加强对崖州历史文化的研究工作，加大崖城孔庙等历史文物保护力度，加快崖城申报国家历史文化名镇的进程，开辟古崖州文化旅游线路；进一步研究和发挥天涯海角、落笔洞、大小洞天等的文化价值，修复一批在全国乃至世界有影响的历史名人故居，五年内在落笔洞建成三亚博物馆，申报一批非物质文化遗产；积极促成妈祖文化生态园建设，力争在五年内建成三亚海洋博物馆、天涯古生物化石博物馆和天涯文化苑。

――加快文化事业发展。推进文化体制改革，完成三亚海滨影剧院改造，充分发挥图书馆的服务作用以及群艺馆、业余体校的培训作用，不断完善现有事业单位的体制功能；突破单一由政府办文化的路子，逐步形成社会文化社会办，国家、集体、个人一起上的新格局；积极建立统一的文化市场，促进文化企业间的合理竞争，创造公平竞争的文化市场环境；进一步完善文化产业价格形成机制，促进文化消费。把文化产业人才培训列入全市人才培训规划，并积极为文化工作者提供优异的策划、创作、生产制作条件，大力支持文化群体开展文化创新活动，吸引外来人才，打造三亚文化人才高地。规划和建设市青少年活动中心、妇女儿童活动中心，进一步推进社区、农村文化站和文化队伍建设；积极开展广场文化活动，做好文化下乡和文体进社区工作，进一步推动全民健身运动发展，丰富群众文体生活，增强群众身体素质。

――积极培育和发展文化产业。落实《关于加快三亚文化产业发展的决定》，坚持实施“文化铺垫、活动支撑、品牌牵引、传媒放大、项目推进、市场运作”的城市品牌发展战略，着手编制《三亚市文化产业发展规划》，合理布局、整合资源，积极发展演艺娱乐、文化旅游、信息网络、美丽时尚、影视制作、动漫、新闻图书出版等产业，重点培育发展一批综合性文化产业集团，使文化产业成为我市又一支柱产业。在金融、财政、税收和土地等方面对重点文化项目建设给予一定的优惠和支持，大力吸引投资，积极促进鲁能“美丽城”会展和高端演艺中心、南中国影视文化生态园、演艺梦幻城、实景山水演艺等项目建设，力争未来五年文化产业项目投资总额超过50亿元；围绕“三亚城市名片”主题活动积极组织实施“一张名片、一部电影、一首歌曲、一部小说、一部风光片、一台文艺节目”的“六个一”文化精品工程，支持组建和引进歌舞表演等文艺团体，特别是要精心组织一台能够反映三亚美丽风光与浪漫风情的大型歌舞节目，努力将其打造成一台在中国旅游界具有较高知名度的大型文艺节目。加快健康体育产业发展，积极推进体育会展中心建设，创造条件建设国家体育冬训基地；全力推动凤凰、半岭温泉、南田旅游区温泉项目实施，努力打造国内外知名的、富有特色的中医保健、康复疗养中心。在继续办好新丝路中国模特大赛、三亚国际广告艺术节、三亚国际美食节、三亚国际铁人三项赛、亚洲高尔夫巡回赛三亚公开赛等传统节庆赛事活动的同时，积极创办首届世界先生大赛、三亚国际沙滩音乐节、国际自行车赛、国际龙舟赛、国际女性电影节等重大节庆赛事活动，进一步塑造“魅力三亚”的城市品牌，为美丽三亚插上文化的翅膀。

五、统筹经济与社会发展，全力打造和谐三亚

要推动社会事业全面进步，促进经济社会协调发展，切实维护群众切身利益，保持社会稳定，促进社会和谐。

――大力发展科教事业。完善科技创新体系，鼓励企业创新，大力推进三亚南繁科学技术研究院、中国南方海洋研究中心、中国三亚农业科学城以及万亩超级水稻基地、反季节西甜瓜设施栽培技术示范基地等建设；积极推进电子政务、电子商务、电子社区、远程教育等信息技术应用。要加快教育资源的合理布局和结构调整，推动优质教育资源共享，促进基础教育均衡发展；进一步加大教育事业投入，特别要加强农村教育基础设施建设，不断完善全市中小学校的教学设施；进一步加大教师培训力度，积极推进教育改革，鼓励社会资本投资教育事业；继续落实“两免一补”政策，扩大公办学校义务教育阶段贫困家庭学生寄宿生活费补助范围，保障农民工子女接受义务教育；采取财政补贴等办法用三年时间解决600户教师住房问题；高质量、高水平普及九年义务教育，并创造条件普及高中阶段教育，全市高中阶段毛入学率达到60%以上；力争在3―5年的时间内，使我市的基础教育、高中教育质量达到全省一流水平；大力发展职业教育和职业技能培训，积极整合资源建设海南规模较大、档次较高、在琼南具有相当影响的三亚市中等职业技术学校，加强适用技术人才培养；继续推进三亚航空旅游职业学院二期和三亚学院二、三期工程建设，积极支持琼州大学迁址三亚，进一步加快高等教育发展，力争五年内全市在校大学生总数达到5万人左右。建立健全人才培养、引进、使用和激励机制，继续加强引进国外智力和派出培训工作，积极构筑人才高地。

――积极推进卫生等社会事业发展。加大投入力度，进一步完善公共卫生和医疗服务体系，继续加快市人民医院改扩建、中医院新区等工程建设，搬迁并做大做强市妇幼保健院，全面提高医疗服务水平，力争将市人民医院建成现代化三级甲等医院，将三亚建成海南南部的医疗中心；理顺医药卫生行政管理体制，加快推进医院采购制度和医疗服务价格改革；继续推进社区医疗服务站的建设，加强药品供应和安全监管网络建设，健全应对突发公共卫生事件机制；深入开展爱国卫生运动，实行市级领导包干街道卫生责任制，力争早日把我市创建成为国家卫生城市；进一步稳定低生育水平，加强性别比综合治理，提高人口质量。广泛开展国防教育，大力支持国防建设，加强国防动员和民兵预备役工作，深入开展拥军优属、拥政爱民活动，提高双拥共建质量，促进军地和谐，努力争创全国双拥模范城。继续做好妇女儿童、科协等工作，外事侨务、民政、档案、修志、统计等工作也要取得新进展。

――加大就业和社保工作力度。实施积极的就业政策，通过壮大经济和产业实力拓宽就业和再就业渠道，利用再就业优惠政策扩大就业容量，力争到2011年全市累计新增就业岗位6万个，城镇登记失业率控制在3%以内；加快户籍和劳动就业制度改革，完善人员流动政策，加快市人才劳动力市场项目建设；进一步落实好税费减免、小额担保贷款、资金奖励和社会保险补贴等一系列优惠政策，鼓励下岗失业人员、高校毕业生、退役军人和其他新增劳动力自主创业和自谋职业。加强劳动保障监察，维护劳动者特别是农民工的合法权益。力争到2011年全市五项社会保险基金征缴率均超过85%；加强社保基金监管，确保社保待遇及时落实；加快推进企业退休人员社会化管理服务工作；进一步完善城市居民最低生活保障和社会弱势群体的福利制度，建立健全新型社会救助体系，将进城务工人员纳入社会保障体系；全面推行农村最低生活保障制度，积极探索新型农村养老保障体系。抓好住房公积金管理，扩大住房公积金缴存的覆盖面，加快经济适用房开发建设，逐步推行城镇廉租房制度，解决中低收入家庭住房难问题。积极发展以扶老、助残、救孤、济困为重点的社会福利事业，加快红十字事业发展。

――加强精神文明和民主法制建设。树立以“八荣八耻”为主要内容的社会主义荣辱观，倡导爱国、敬业、诚信、友善等道德规范；加强科普工作，倡导科学、健康、文明的生活方式，大力推行城乡居民文明规范，提高居民文明水平和城乡文明程度。充分发挥广播电视、报刊、互联网站等媒体的舆论导向作用，提高舆论引导水平。广泛开展和谐创建活动，把和谐家庭、和谐社区、和谐企业、和谐行业等和谐创建活动同群众性精神文明创建活动结合起来，推动形成人人促进和谐的局面。要坚持和完善人民代表大会制度和共产党领导的多党合作、政治协商制度；自觉向人大及其常委会报告工作，接受人大的法律监督和工作监督，支持和保证人大及其常委会依法行使各项权力；充分尊重人民政协以及各民主党派在参政议政中的作用，自觉接受其民主监督；认真做好人大议案、建议和政协提案的办理工作；积极加强同工商联、无党派人士的团结合作，充分支持工会、共青团、妇联、文联、侨联等人民团体发挥桥梁纽带作用。积极稳妥有序地推进基层民主政治建设，搞好居委会换届选举工作，完善政务公开制度，切实保证人民群众的知情权、参与权、表达权、监督权。加强司法民主建设，进一步支持审判公开、检务公开。加强司法队伍建设，不断提高司法人员素质。做好普法工作，引导全社会学法、知法、守法、用法，不断提高全民法律素质。加强对行政机关、司法机关的监督，促进依法行政、公正司法。

――切实维护社会稳定。抓好平安三亚创建，力争在3―5年时间内，将三亚打造成全国最平安的城市之一。进一步加强社会治安综合治理，严厉打击各种有组织犯罪、黑恶势力犯罪和经济犯罪活动；继续深入开展打击“两抢一盗”和毒品犯罪等专项行动，加大“无毒市”建设力度；积极实施“天网工程”，推广电子警察使用，提高监控科技水平。加强安全生产工作，健全安全管理体系，强化各级安全责任制的落实，减少一般事故，坚决遏制重特大安全事故的发生；建立健全台风、地震、海啸、海事、重大旱涝灾害以及食品药品、公共卫生等重大公共安全事件的预警、应急、救援体系，加快公共安全抢险救援应急中心建设，提高政府应对自然灾害和公共危机的能力。积极推行领导干部接待群众制度，进一步落实信访责任制，建立健全社会利益协调机制和社会矛盾纠纷调处机制，预防和妥善处置群体性事件。大力推进社区建设，引导社区中介组织有序、规范运作，健全新型基层社会管理和服务体制，实现政府行政管理和社区自我管理的有效衔接。全面贯彻党的民族宗教政策，加大对少数民族地区发展的支持力度，依法管理宗教事务，防止境外敌对势力和非法宗教势力的渗透。

各位代表！时代赋予我们光荣而神圣的使命，重担在肩，任重道远。让我们更加紧密地团结在以胡锦涛同志为总书记的党中央周围，在市委的坚强领导下，以饱满的热情、昂扬的斗志，脚踏实地、开拓创新、奋发图强，为推动三亚又好又快发展，早日把三亚建设成为国际性热带滨海旅游城市而努力奋斗！
